# Inulin from Jerusalem artichoke tubers alleviates hyperglycaemia in high-fat-diet-induced diabetes mice through the intestinal microflora improvement

**DOI:** 10.1017/S0007114519002332

**Published:** 2020-02-14

**Authors:** Tianyun Shao, Qiuhong Yu, Tingshuo Zhu, Anhong Liu, Xiumei Gao, Xiaohua Long, Zhaopu Liu

**Affiliations:** College of Resources and Environmental Sciences, Nanjing Agricultural University, Nanjing, Jiangsu 210095, People’s Republic of China

**Keywords:** Jerusalem artichokes, Inulin, Hyperglycaemia, Enteric micro-organisms, Lipid genes

## Abstract

The rate of hyperglycaemia in people around the world is increasing at an alarming rate at present, and innovative methods of alleviating hyperglycaemia are needed. The effects of Jerusalem artichoke inulin on hyperglycaemia, liver-related genes and the intestinal microbiota in mice fed a high-fat diet (HFD) and treated with streptozotocin (STZ) to induce hyperglycaemia were investigated. Inulin-treated hyperglycaemic mice had decreased average daily food consumption, body weight, average daily water consumption and relative liver weight and blood concentrations of TAG, total cholesterol, HDL-cholesterol and fasting blood glucose. Liver-related gene expressions in hyperglycaemic (HFD-fed and STZ-treated) compared with control mice showed eighty-four differentially expressed genes (forty-nine up-regulated and thirty-five down-regulated). In contrast, hyperglycaemic mice treated with inulin had twenty-two differentially expressed genes compared with control ones. Using Illumina high-throughput sequencing technology, the rarefaction and the rank abundance curves as well as the *α* diversity indices showed the treatment-induced differences in bacterial diversity in intestine. The linear discriminant analysis of effect size showed that the inulin treatment improved intestinal microbiota; in particular, it significantly increased the number of *Bacteroides* in the intestine of mice. In conclusion, inulin is potentially an effective functional food for the prevention and/or treatment of hyperglycaemia.

Nowadays, diabetes is a major health problem in developed as well as in developing countries. The proportion of diabetes in the population has increased year by year, becoming the third most serious disease after CVD and cancer. Diabetes is accompanied by cardiovascular complications. It is very difficult to predict the development of disease^(1)^. The prediction is that the world’s population of type 2 diabetes will reach 366 million in 2030^(2)^. A complete or relative lack of insulin secretion and/or action can lead to diabetes and its complications^(3)^. There is a close association between diabetes and hyperlipidaemia and hypercholesterolaemia^(4–4)^. In addition, recent evidence suggests that changes in haematological parameters may lead to abnormal glucose metabolism and diabetes mellitus through increasing insulin resistance and liver dysfunction^(7,8)^.

Intestinal microbiota plays an important role in the development of inflammation and metabolic disorders of obesity, insulin resistance and type 2 diabetes^(9)^. The intestinal microbiota is considered to be a prescribed indicator for the management of type 2 diabetes and the prevention of other microscopic and macroscopic vascular diseases^(10)^. The intestinal microbiota may be related to the production of lipopolysaccharides and metabolic endotoxaemia^(11)^. Probiotics can restore intestinal microbiota of *Akkermansia muciniphila* in diabetics and obese subjects. Inulin can produce changes in intestinal bifidobacteria^(12–14)^ and *Bacteroides* as well as reduce the abundance of Firmicutes^(15–17)^.

Jerusalem artichoke (*Helianthus tuberosus* L.) is a perennial tuber plant. Its tubers are made largely of carbohydrates, mainly in the form of inulin. Inulin is soluble fibre and contains a short chain of fructose molecules as *β*-2,1 fructan. Streptozotocin (STZ) is a toxic nitrosourea analogue, and it can selectively destroy specific pancreatic *β* cells via GLUT2 in mice and rats^(18,19)^. STZ can inhibit the function of pancreatic *β* cells and reduce the secretion of insulin. Hence, it is often used to establish the animal model of diabetes. Low-dose STZ can slightly damage the function of pancreatic *β* cells and can moderately reduce insulin secretion, creating symptoms similar to those in patients with type 2 diabetes insulin hyposecretion^(20)^.

In the present study, a repeatable experimental model of hyperlipidaemia–diabetic mice was induced by feeding a high-fat diet (HFD) for 1 consecutive month and multiple low doses (50 mg/kg) of STZ for 1 week. Considering Jerusalem artichoke inulin’s potential health benefits, the present trial aimed to investigate its effects on fasting blood glucose (FBG) homoeostasis, average daily food consumption, body weight, average daily water consumption, relative liver weight, serum lipids level, liver-related gene expressions and intestinal microbiota in hyperlipidaemia–diabetes mice.

## Material and methods

### Experimental material and chemicals

Inulin (extracted from Jerusalem artichoke tubers) was purchased from the Qinghai Weide Biotechnology Co., Ltd.

The total sugar content was determined using the phenol–sulphuric acid method. The purity of inulin was determined by subtracting the reducing sugar content from the total sugar content (3-amino-5-nitrosalicylic acid method)^(21,22)^. The purity of inulin was 90·1 %.

Metformin HCl (1,1-dimethyl metformin HCl, C_14_H_11_N_5_.HCl, molecular weight 165·63 g/mol) tablets were purchased from Sino-American Shanghai Squibb Pharmaceuticals Ltd. Metformin HCl is a biguanide antihyperglycaemic drug administered orally alone or in combination with other hypoglycaemics in treating type 2 diabetes mellitus^(23–26)^. STZ (C_8_H_15_N_3_O_7_, molecular weight 265·22 g/mol) was purchased from the Biosharp Co., Ltd).

### Ethics statement of animal experiments

All of the procedures involving mice were carried out in accordance with the Guidelines for the Care and Use of Laboratory Animals prepared by the Institutional Animal Care and Use Committee of Nanjing Agricultural University, Nanjing, China (SYXK(SU)2017-0007). The experimentation was performed in the laboratory animal centre of Nanjing Agricultural University. The method of euthanasia was cervical dislocation.

### Experimental animals and diets

The feeding method of Yu *et al.*^(27)^ was followed. Sixty male 6-week-old C57BL/6J mice were provided by the Yangzhou University Medical Center; each weighed about 20 g (the experimental animal production license: SCXK (Su) 201605253). All animals had free access to drinking water and were fed a standard diet for 7 d and then followed by feeding them with the standard (control) diet or HFD MD1203 (45 % energy from fat) purchased from Medicience Ltd. (Yangzhou, China). The high-fat with a high-sucrose diet is an effective method to induce insulin resistance. The detailed composition of the diet is shown in Table 1. Mice were housed in plastic cages (five per cage) under standard conditions (20–26°C, 40–70 % relative humidity, 12 h light–12 h dark cycle). The standard (control) diet was provided by the Animal Experimental Center of Nanjing Agricultural University, and its formula is formulated according to the ‘Experimental Animal Compound Feed Nutritional Component (GB14924.3-2010)’ in the National Standard for Experimental Animal Environment and Facilities (GB14925-2010). The comparison of nutrients between the standard (control) diet and HFD is shown in online Supplementary Table S1.

**Table 1.** Composition of the high-fat diet

Name of component	Percentage
Casein	23·31
l-Cysteine	0·35
Maize starch	8·48
Malt	11·65
Sucrose	20·14
Cellulose	5·83
Soyabean oil	2·91
Lard	20·68
Composite minerals	5·23
Compound vitamins	1·16
Choline bitartate	0·23
Total heat (kJ/g)	19·78

### Development of high-fat-diet-fed and streptozotocin-treated type 2 diabetic mice

In order to investigate the alleviation effect of inulin on the type 2 diabetes, mice were fed the HFD and treated with STZ (Table 2). According to a power calculation to determine sample size, the sixty C57BL/6J mice were randomly divided into two groups: the normal control group (*n* 10) and the experimental group (*n* 50); the normal control group was fed the standard (control) diet, and the experimental group was fed the HFD for 4 weeks. Then the experimental group was injected intraperitoneally with low-dose STZ (50 mg/kg, in citrate buffer, pH 4·4) for 1 week, whereas the control group mice were given just the same volume of citrate buffer preparation. After another 2 weeks of feeding, all the mice were fasted for 12 h, and the FBG test was carried out by ACCU-CHEK Active (Roche Diagnostics GmbH). FBG ≥ 11·1 mmol/l is the diagnostic criterion for diabetes. All mice had unlimited access to drinking water.

**Table 2.** Construction of experimental mouse model*

Standard (control)		High fat	
Treatments	Time	Treatments	Time
Standard (control) diet	1 week	Standard (control) diet	1 week
Overnight fasting	12 h	Overnight fasting	12 h
Standard (control) diet	4 weeks	High-fat diet	4 weeks
Citrate acid buffer	1 week	STZ	1 week
Standard (control) diet	2 weeks	High-fat diet	2 weeks
Overnight fasting	12 h	Overnight fasting	12 h
Intra-gastric administration (forenoon)	4 weeks	Intra-gastric administration (forenoon)	4 weeks
Overnight fasting	12 h	Overnight fasting	12 h

STZ, streptozotocin.

* All mice had unlimited access to drinking water.

### Experimental design

The experimental setup consisted of six different groups. Type 2 diabetic mice were randomly divided into five groups (ten mice per group) and treated daily with intra-gastric administration (0·2 ml/10 g) of each test compound (as described in the previous section) for 4 weeks. The solvent was made with distilled water. Then we fed group CK mice (blank control, standard diet + 5 g/kg physiological saline per d) and group H mice (experimental control group with induced diabetes, standard diet + 5 g/kg physiological saline per d) the standard diet, and they received intra-gastric administration of 5 g/kg physiological saline (0·9 % NaCl w/v) per d for 4 weeks. Group CP mice (positive control group, standard diet + metformin HCl tablets 125 mg/kg per d) were fed the standard diet and treated with 125 mg/kg per d metformin HCl tablets. Mice in groups LJ (standard diet + inulin 2·5 g/kg per d), MJ (standard diet + inulin 5 g/kg per d) and HJ (standard diet + inulin 10 g/kg per d) were fed the standard diet and treated with, respectively, 2·5, 5 and 10 g inulin/kg per d (the inulin irrigation amounts were, respectively, 5, 10 and 20 times the recommended daily intake for people)^(28)^.

### Determination of weekly body weight and relative liver weight

The Yu’s^(27)^ feeding method was followed. Body weight of each mouse was measured weekly. At the end of the experiment, the relative liver weight per 100 g of total weight of each mouse was calculated:
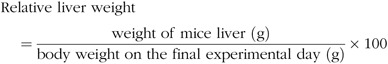



### Blood collection and biochemical assays

At the end of the 4-week treatment, all the mice were fasted for 12 h and the FBG test was carried out by ACCU-CHEK Active. The blood samples were collected from the orbital sinus of mice using a 2-ml heparinised syringe and placed on ice for transfer and then centrifuged at 3500 ***g*** at 4°C for 15 min to separate the plasma. The concentrations of total cholesterol, TAG, HDL-cholesterol and LDL-cholesterol in plasma were measured using a Roche MODULAR automatic biochemical analyser at the Integrated Traditional Chinese and Western Medicine Hospital of Nanjing University of Chinese Medicine (Nanjing, China).

### Liver collection, total RNA extraction, reverse transcription and real-time PCR

The liver was taken out, fat cleaned and the liver flushed by physiological saline to remove the surface blood at 4°C. The related gene expression of liver was performed at Shanghai Wcgene Biotech Co. Ltd.

Briefly, to isolate the total RNA, approximately 30 mg of the main lobe from each liver was placed into RNAiso Plus (Takara Co. Ltd), according to the manufacturer’s instructions and then resuspended in diethyl pyrocarbonate-treated water. The quality of RNA was assessed by electrophoresis in 1·0 % (w/v) formaldehyde denaturing agarose gel. Real-time PCR was used to determine mRNA levels based on the Roche FS Universal SYBR Master: 04913914001 instructions in an ABI ViiA7 Real-Time PCR System. A 20-μl reaction system contained 5 μl Roche FS Universal SYBR Master (ROX), 3 μl ddH_2_O, 0·75 μl Primer F, 0·75 μl Primer R, 0·5 μl DNA sample and 10 μl total volume. The cycling parameters were as follows: 10 min at 95°C followed by one cycle, 30 s at 95°C followed by forty cycles, 30 s at 60°C followed by forty cycles and 10 min at 72°C followed by one cycle.

### DNA extraction and intestinal micro-organism communities

Three replicate mice were randomly selected from each experimental group. Total bacterial DNA from 0·25 g of colon segment of the large intestine was extracted using a PowerFecal^TM^ DNA Isolation kit (MO BIO Laboratories Inc), according to the manufacturer’s instructions and was stored at −80°C for further analysis^(29)^. Amplicon pyrosequencing was performed on an Illumina MiSeq platform at Allwegene Technology Inc.

Briefly, DNA was amplified using the 338F/806R primer set (338F: 5′-ACTCCTACGGGAGGCAGCAG-3′, 806R: 5′-GGACTACHVGGGTWTCTAAT-3′) that targets the regions (V3–V4) of the 16S rRNA gene because sequences in those regions provided the greatest diversity at the domain and phylum levels. The PCR was performed in 25-μl volume containing 30 ng of DNA template. The cycling parameters were as follows: 5 min of denaturation at 95°C followed by twenty-five cycles of 30 s at 95°C, 30 s for annealing at 56°C and 40 s at 72°C (elongation), with a final extension at 72°C for 10 min. PCR products were processed with an AxyPrep™ Mag PCR Normaliser kit (Axygen, Inc.) for normalisation.

Quantitative Insights Into Microbial Ecology (https://qiime.org/tutorials/processing_illumina_data.html) quality filters were used to filter the reads^(30)^. We used the Cluster Database at High Identity with Tolerance (CD-HIT) pipeline for picking operational taxonomic units (OTU) and making OTU table. The sequences with similarity of 97 % were assigned to OTU. A representative sequence was selected for each OTU, and the classification data were assigned to each of the representative sequences using the Ribosome Database Project (RDP) classifier^(31)^.

To estimate *α* diversity, the OTU table was rarefied, and four indicators were calculated: Chao 1 estimating richness, the observed OTU as the only OTU counting sample, and Shannon index estimating diversity^(32,33)^. Each sample was classified and statistically analysed using RDP, Greengene and Silva three database comparison^(34–36)^. The sequences obtained were distributed in eight bacterial phyla by RDP (V14 https://rdp.cme.msu.edu/).

Linear discriminant analysis (LDA) and effect size measurement (LEfSe) are common methods for the discovery of macrogenomic biomarkers. We performed LEfSe calculations for the non-parametric Wilcoxon sum-rank test followed by LDA using online software (https://huttenhower.sph.harvard.edu/galaxy/) to assess the effect size of each taxon with differential abundance^(37)^.

The complete datasets were deposited in the National Center for Biotechnology Information. The Sequence Read Archive accession number is SRP108873.

### Statistical analysis

Data were analysed by one-way ANOVA using SPSS 19.0 (SPSS Inc.). All data were tested for homogeneity of variance by Levene’s test. Tukey’s test was then used to compare the differences among the treatments. The level of significance was set at *P* ≤ 0·05. All data were expressed as means with their standard errors.

## Results

### The high-fat-diet-fed and streptozotocin-treated mice showed type 2 diabetes

No difference in the concentration of FBG was observed between these experimental mice before the induction (online Supplementary Table S2). After the induction of type 2 diabetes (the samples were taken after 2 weeks after diabetes induction), the FBG concentration in the HFD-fed and STZ-treated mice (treatment H, experimental control) increased significantly (*P* ≤ 0·05) (FBG ≥ 11·1 mmol/l) compared with the blank control (CK, standard diet and citrate buffer vehicle) mice. Hence, the treatment with HFD feed and STZ successfully induced type 2 diabetes in mice.

### Effect of inulin on average daily food consumption, body weight, average daily water consumption and relative liver weight in high-fat-diet-fed and streptozotocin-treated type 2 diabetic mice

Metformin HCl tablets (treatment CP, positive control), inulin of 2·5 g/kg per d (treatment LJ), inulin of 5 g/kg per d (MJ group) or inulin of 10 g/kg per d (treatment HJ) was orally administered to the HFD-fed and STZ-treated type 2 diabetic-induced mice for 4 weeks. Body weight decreased significantly (*P* ≤ 0·05) in the H (experimental control), CP, LJ, MJ and HJ groups compared with the CK group (Table 3). Body weight decreased in a dose-dependent way. Average daily food consumption and average daily water consumption were decreased in a dose-dependent manner in the LJ, MJ and HJ groups (Table 3). Compared with the H group, relative liver weight decreased significantly (*P* ≤ 0·05) in the groups treated with metformin HCl tablets, inulin of 2·5, 5 or 10 g/kg per d, but no significant difference was observed between the three groups treated with inulin.

**Table 3.** Average daily food consumption, body weight, average daily water consumption and relative liver weight as influenced by treating mice for 4 weeks with standard diet and orally with metformin HCl tablets or intra-gastrically with three different concentrations of inulin* (Mean values with their standard errors)

	Average daily food consumption (g/d)		Body weight after 4-week treatment (g)		Average daily water consumption (ml/d)		Relative liver weight (%)	
Treatment group	Mean	se	Mean	se	Mean	se	Mean	se
CK†	3·15^c^	0·12	25·20^a^	0·24	3·57^c^	0·23	4·64^d^	0·11
H‡	3·15^c^	0·06	23·49^c^	0·23	14·13^a^	0·57	7·35^a^	0·36
CP§	4·52^a^	0·14	24·41^b^	0·18	13·78^a^	0·42	6·11b	0·11
LJ||	4·68^a^	0·13	24·08^b^	0·21	15·30^a^	0·79	5·58^b,c^	0·14
MJ¶	4·47^a^	0·11	23·37^c^	0·17	14·31^a^	0·38	5·43^c^	0·23
HJ**	3·89^b^	0·15	22·70^d^	0·17	10·58^b^	0·43	5·48^c^	0·14

^a,b,c,d^ Mean values in a column with unlike superscript letters are significantly different (*P* ≤ 0·05).

* Inulin (isolated from Jerusalem artichoke tubers) is a short-chain polymer of fructose molecules containing a high concentration of fructan.

† CK, standard diet + physiological saline of 5 g/kg per d (blank control).

‡ H, standard diet + physiological saline of 5 g/kg per d (experimental control group with induced diabetes).

§ CP, standard diet + metformin HCl tablets of 125 mg/kg per d.

|| LJ, standard diet + inulin of 2·5 g/kg per d.

¶ MJ, standard diet + inulin of 5 g/kg per d.

** HJ, standard diet + inulin of 10 g/kg per d.

### Effect of inulin on serum lipid levels and FBG in high-fat-diet-fed and streptozotocin-treated type 2 diabetic mice

Compared with group H, the serum concentrations of total cholesterol, TAG, HDL-cholesterol and FBG in the CP, LJ, MJ and HJ groups decreased to a similar degree (between 27 and 70 %) (Table 4). In the LJ, MJ and HJ groups, the serum concentrations of HDL-cholesterol and FBG decreased in a dose-dependent manner. In contrast, the serum concentration of LDL-cholesterol varied, that is, being the same in the H, CP, LJ, and HJ groups but lower in the MJ group compared with the H group (Table 4).

**Table 4.** Effect of 4 weeks of the oral treatment with metformin HCl tablets or the intra-gastric treatment with three different concentrations of inulin on serum lipid levels and fasting blood glucose in experimental mice with induced type 2 diabetes (Mean values with their standard errors)

	Concentration (mmol/l)									
	Total cholesterol		TAG		HDL-cholesterol		LDL-cholesterol		Fasting blood glucose	
Treatment group	Mean	se	Mean	se	Mean	se	Mean	se	Mean	se
CK*	2·03^c^	0·08	1·01^b^	0·05	1·89^c^	0·05	0·32^c^	0·03	7·09^c,d^	0·19
H†	4·40^a^	0·28	2·69^a^	0·21	2·81^a^	0·18	0·99^a^	0·11	17·62^a^	1·09
CP‡	3·12^b^	0·13	1·15^b^	0·07	2·28^b^	0·12	0·91^a^	0·04	10·19^b^	0·96
LJ§	2·99^b^	0·10	1·23^b^	0·09	2·26^b^	0·04	0·80^a,b^	0·04	11·39^b^	1·31
MJ||	2·86^b^	0·18	1·21^b^	0·06	2·12^b,c^	0·12	0·69^b^	0·07	9·55^b,c^	1·06
HJ¶	2·78^b^	0·07	1·01^b^	0·05	1·95^c^	0·03	0·93^a^	0·07	5·29^d^	0·38

^a,b,c,d^ Mean values in a column with unlike superscript letters are significantly different (*P* ≤ 0·05).

* CK, standard diet + physiological saline of 5 g/kg per d (blank control).

† H, standard diet + physiological saline of 5 g/kg per d (experimental control group with induced diabetes).

‡ CP, standard diet + metformin HCl tablets of 125 mg/kg per d.

§ LJ, standard diet + inulin of 2·5 g/kg per d.

|| MJ, standard diet + inulin of 5 g/kg per d.

¶ HJ, standard diet + inulin of 10 g/kg per d.

### Liver-related gene expression in hyperglycaemic mice

**Fig. 1.**
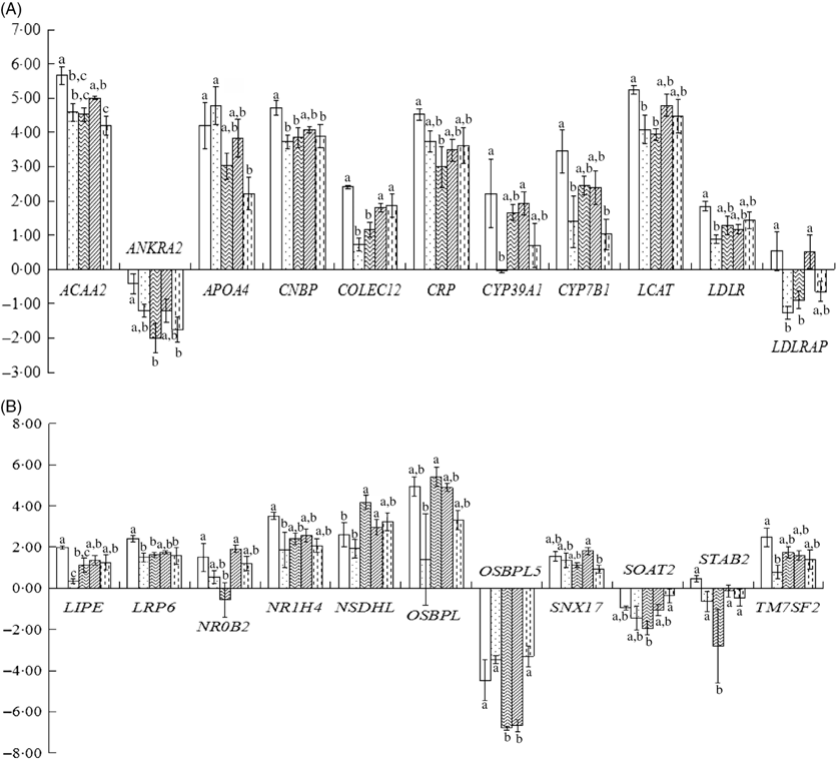
Liver-related gene expression in hyperglycaemic mice. (A) and (B) Liver-related gene expression. CK, standard diet + physiological saline of 5 g/kg per d (blank control); H, standard diet + physiological saline of 5 g/kg per d (experimental control group with induced diabetes); CP, standard diet + metformin HCl tablets of 125 mg/kg per d; LJ, standard diet + inulin of 2·5 g/kg per d; HJ, standard diet + inulin of 10 g/kg per d. ^a,b,c^ Mean values for a gene with unlike letters are significantly different (*P* < 0·05). 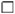, CK; 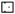, H; 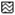, CP; 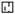, LJ; 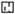, HJ.

As shown in Fig. 1(A) and (B), treatment differences were found in the expression of twenty-two up- and down-regulated genes such as *ACAA2*, *ANKRA2*, *APOA4*, *CNBP*, *COLCE12*, *CRP*, *CYP39A1*, *CYP7B1*, *LCAT*, *LDLR*, *LDLRAP1*, *LIPE*, *LRP6*, *NR0B2*, *NR1H4*, *NSDHL*, *OSBPL1A*, *OSBPL5*, *SNX17*, *SOAT2*, *STAB2* and *TM7SF2*, though the differences were not always significant.

### Diversity and richness indices of intestinal microbiota

Four diversity indices showed a similar trend. The OTU and phylogenetic diversity (PD) whole tree and Shannon indices of the CP and HJ groups were significantly different from the LJ treatment group (*P* ≤ 0·05) (Table 5).

**Table 5.** Comparison of estimated operational taxonomic unit (OTU) richness and diversity indices (*α* diversity index) of the mouse intestinal 16S rDNA gene libraries for clustering at 97 % identity as obtained from pyrosequencing analysis (Mean values with their standard errors)

	Chao 1 index	OTU	PD whole tree index	Shannon index
CK*	268^a,b^	239^a,b^	18·8^a,b^	5·53^a,b^
H†	255^a,b^	234^a,b^	19·4^a,b^	5·78^a^
CP‡	226^b^	188^b^	16·6^b^	4·87^b,c^
LJ§	299^a^	267^a^	21·1^a^	5·67^a^
HJ||	238^a,b^	201^b^	17·3^b^	4·73^c^

PD, Phylogenetic diversity.

^a,b,c^ Mean values in a column with unlike superscript letters are significantly different (*P* ≤ 0·05).

* CK, standard diet + physiological saline of 5 g/kg per d (blank control).

† H, standard diet + physiological saline of 5 g/kg per d (experimental control group with induced diabetes).

‡ CP, standard diet + metformin HCl tablets of 125 mg/kg per d.

§ LJ, standard diet + inulin of 2·5 g/kg per d.

|| HJ, standard diet + inulin of 10 g/kg per d.

**Fig. 2.**
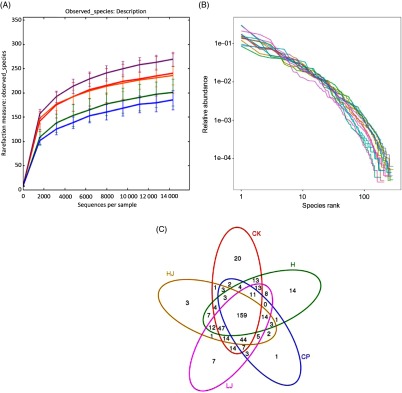
(A) Rarefaction curves showing the observed species (operational taxonomic units; OTU) richness (97 % identity) of the 16S rDNA gene with increasing sequencing depth. (B) Rank abundance curves showing the richness and evenness of the observed species (97 % identity) based on the 16S rDNA gene. (C) Venn diagram depicting OTU of bacteria detected in mice intestinal contents as influenced by the treatments. CK, standard diet + physiological saline of 5 g/kg per d (blank control); H, standard diet + physiological saline of 5 g/kg per d (experimental control group); CP, standard diet + metformin HCl tablets of 125 mg/kg per d; LJ, standard diet + inulin of 2·5 g/kg per d; HJ, standard diet + inulin of 10 g/kg per d. 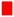, CK; 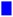, CP; 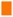, H; 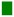, HJ; 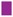, LJ. 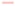, CK1; 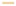, CK2; 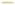, CK3; 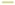, H1; 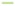, H2; 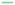, H3; 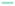, CP1; 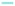, CP2; 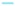, CP3; 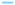, LJ1; 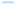, LJ2; 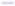, LJ3; 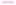, HJ1; 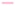, HJ2; 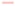, HJ3.

The number of OTU was lower in the CP group compared with the other four treatment groups (except HJ, Fig. 2(A)). The distribution of the species did not correspond with an increase in the OTU (Fig. 2(B)). In the Venn diagram, the number of unique sequences was largest in the CK (20) and smallest in CP (1) groups, and 159 in common among the five treatment groups (Fig. 2(C))^(38)^.

### Statistical analysis of species classification

**Fig. 3.**
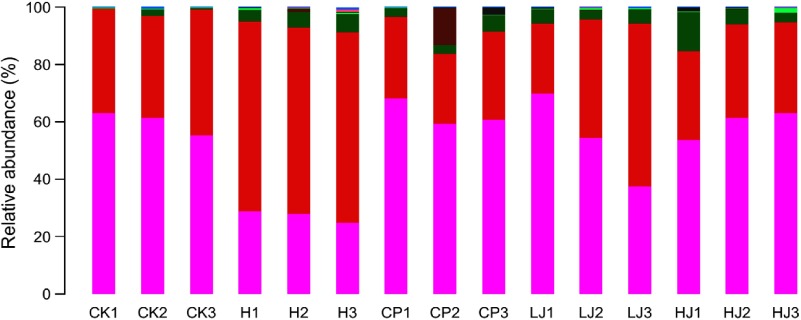
Relative abundance of the dominant bacterial phyla in mouse intestinal contents as influenced by the treatments. The relative abundances are based on the proportional frequencies of DNA sequences that could be classified at the phylum level. CK, standard diet + physiological saline of 5 g/kg per d (blank control); H, standard diet + physiological saline of 5 g/kg per d (experimental control group); CP, standard diet + metformin HCl tablets of 125 mg/kg per d; LJ, standard diet + inulin of 2·5 g/kg per d; HJ, standard diet + inulin of 10 g/kg per d. 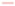, p__Bacteroidetes; 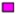, p__Firmicutes; 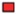, p__Proteobacteria; 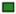, p__Verrucomicrobia; 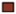, p__Actinobacteria; 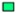, p__Cyanobacteria; 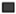, p__Candidate_division_TM7; 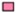, other.

**Fig. 4.**
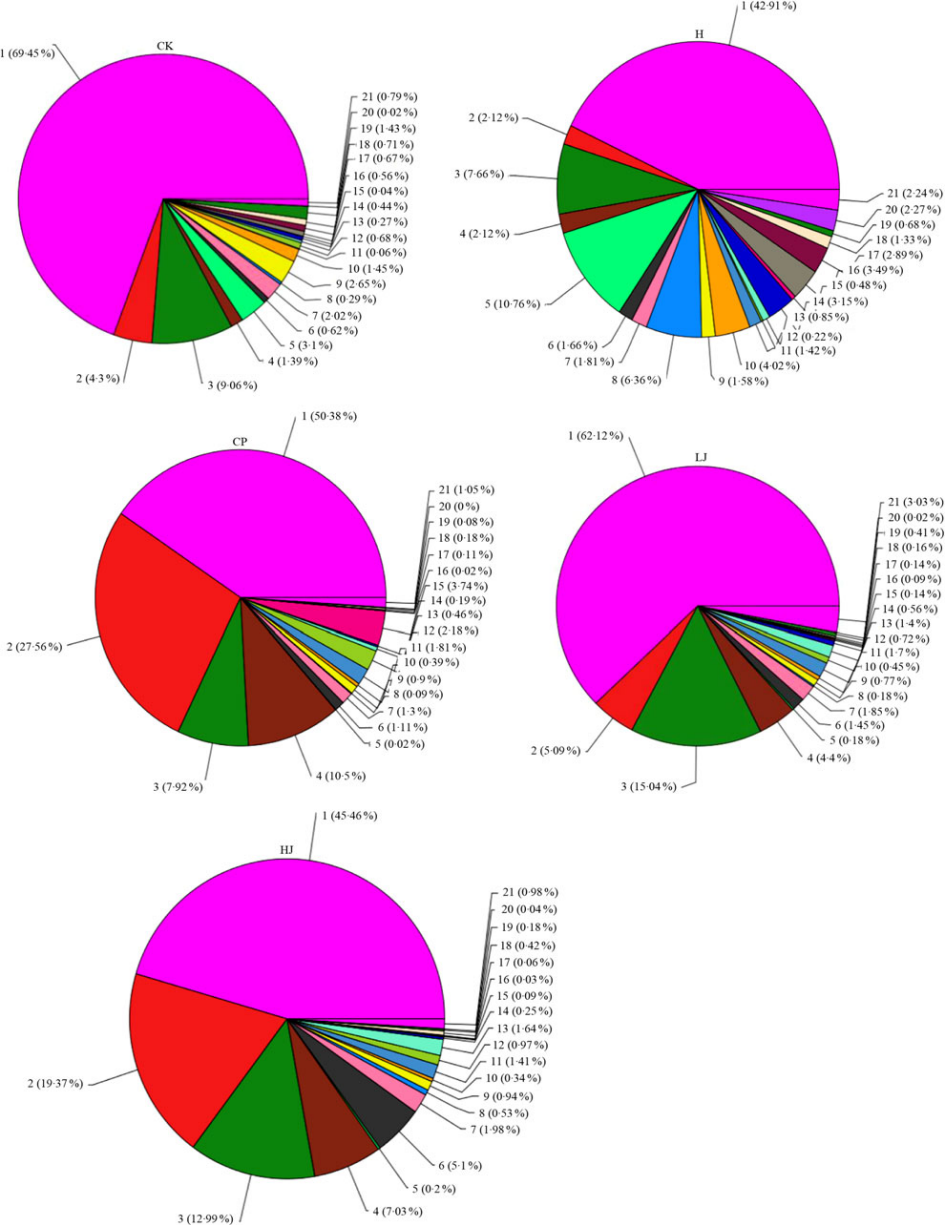
Percentage of different bacterial families in each sample. Data are expressed as means (*n* 3). Sequences that could not be classified into any known groups were labelled ‘other’. CK, standard diet + physiological saline of 5 g/kg per d (blank control); H, standard diet + physiological saline of 5 g/kg per d (experimental control group); CP, standard diet + metformin HCl tablets of 125 mg/kg per d; LJ, standard diet + inulin of 2·5 g/kg per d; HJ, standard diet + inulin of 10 g/kg per d. 1: g__unidentified; 2: g__Bacteroides; 3: g__Blautia; 4: g__Incertae_Sedis; 5: g__Allobaculum; 6: g__Helicobacter; 7: g__Alistipes; 8: g__Dorea; 9: g__Odoribacter; 10: g__Intestinimonas; 11: g__Bilophila; 12: g__Parabacteroides; 13: g__RC9_gut_group; 14: g__Anaerotruncus; 15: g__Akkermansia; 16: g__Roseburia; 17: g__Oscillibacter; 18: g__Lactobacillus; 19: g__Coprococcus; 20: g__Desulfovibrio; 21: other.

In general, bacterial composition of different samples was similar with regard to the phyla present but varied in the distribution of each phylum (Fig. 3). The sequences obtained were distributed by RDP in sixty-one bacterial taxa. In general, bacterial composition of different samples was similar with regard to the taxa present but varied in the distribution of each taxon (Fig. 4). Unidentified and others represented a large proportion in all groups, accounting for more than 45 % of the reads. Compared with the CK group, the H group had significantly higher proportions of *Incertae sedis, Allobaculum, Helicobacter, Dorea, Intestinimonas, Bilophila,* RC9 gut group, *Anaerotruncus, Akkermansia, Roseburia, Oscillibacter, Lactobacillus* and *Desulfovibrio* (1·5, 3·5, 2·7, 22, 2·8, 24, 3·2, 7·2, 12, 6·2, 4·1, 1·2 and 114 times respectively) and lower proportions of *Bacteroides, Blautia, Alistipes, Odoribacter, Parabacteroides* and *Coprococcus* (0·49, 0·85, 0·90, 0·60, 0·32 and 0·48 respectively). Compared with the H group, the inulin treatment LJ and HJ groups had significantly higher proportions of *Bacteroides* (2·4 and 9·1 times, respectively), *Blautia* (2·0 and 1·7 times, respectively), *Incertae sedis* (2·1 and 3·3 times, respectively), *Helicobacter* (0·9 and 3·1 times, respectively), *Alistipes* (1·0 and 1·1 times, respectively), *Bilophila* (1·2 and 1·0 times, respectively), *Parabacteroides* (3·3 and 4·4 times, respectively) and RC9 gut group (1·7 and 1·9 times, respectively) and lower proportions of *Allobaculum* (0·02 and 0·02, respectively), *Dorea* (0·03 and 0·08, respectively), *Odoribacter* (0·49 and 0·54, respectively), *Intestinimonas* (0·11 and 0·08, respectively), *Anaerotruncus* (0·18 and 0·08, respectively), *Akkermansia* (0·29 and 0·19, respectively), *Roseburia* (0·03 and 0·01, respectively), *Oscillibacter* (0·05 and 0·02, respectively), *Lactobacillus* (0·12 and 0·32, respectively), *Coprococcus* (0·60 and 0·26, respectively) and *Desulfovibrio* (0·01 and 0·02, respectively).

We used LEfSe, a statistical tool designed to find biomarkers in the metagenome data with default parameters, to identify potential discriminating taxa among treatments. LEfSe was performed to obtain the cladogram representation and the predominant bacteria in the intestinal microbiota in the control (CK) and four treatment groups (H, CP, LJ and HJ; online Supplementary Fig. S1(A)). Online Supplementary Fig. S1(B) shows the largest differences in taxa among the four communities. On a genus level, *Incertae sedis* was enriched in the CK group, *Dorea*, *Roseburia*, *Anaerotruncus*, *Oscillibacter*, *Allobaculum,* Candidate division TM7, *Streptococcus*, *Candidatus Saccharimonas*, *Acetatifactor*, *Mucispirillum*, *Peptococcus*, *Anaerovorax*, *Turicibacter* and *Clostridium sensu stricto 1* were enriched in the H group; *Bacteroides*, *Anaerostipes* and *Morganella* were enriched in the CP group; *Acaligenes*, *Ruminococcus*, *Paenalcaligenes*, *Wautersiella* and *Flavonifractor* were enriched in the LJ group, whereas *Incertae sedis* and *Subdoligranulum* were enriched in the HJ group.

## Discussion

According to previous studies, inulin improved insulin secretion, the damaged liver and reduced the levels of blood glucose, serum cholesterol and TAG in the STZ-treated mice with induced diabetes^(39)^. Studies have shown that inulin can increase the number of beneficial bacteria, such as bifidobacteria, lactobacilli and certain butyrate-producing bacteria in the colon, and reduce the population of the harmful bacteria from the *Clostridium perfringens* group^(40,41)^. Similarly, research showed that inulin can improve the levels of cholesterol, blood fat and blood sugar^(42)^. However, the hypoglycaemic effect of inulin needs to be studied in more detail, and the potential mechanisms remain to be determined.

In the HJ group, mice treated with inulin showed a decrease in body weight (Table 3). A hyperglycaemic mouse model was fed the HFD in combination with STZ treatment. After 4 weeks of treatment, the weight of the HJ group was significantly lower compared with the H group (*P* ≤ 0·05) and the weight of the inulin-treated group (LJ and MJ groups). It showed a significant downward trend and a dose dependency. However, compared with the H group, the daily average feed consumption of the CP, LJ, MJ and HJ groups increased significantly (*P* ≤ 0·05). This phenomenon indicates that although the daily consumption of the feed increased after the Jerusalem artichoke inulin treatment, the body weight did not increase. Compared with the H group, the relative liver weight and daily average drinking water consumption of the CP, LJ, MJ and HJ groups were reduced, though this reduction may not be significant, and the higher the relative liver weight ratio, the higher the utilisation of fat components in the feed^(43)^. Such a result was unlikely caused by appetite suppression; on the contrary, the energy consumption of inulin-treated animals may increase. Relative liver weight decreased significantly (*P* ≤ 0·05) in the treatment groups compared with the H group (Table 3). A high relative ratio of liver weight shows significant amounts of fat deposited in the liver^(39)^.

Importantly, inulin was more effective than metformin HCl tablets in decreasing total cholesterol levels in hyperglycaemic mice (Table 4). The FBG and serum lipid levels in the mice of the Jerusalem artichoke inulin group were significantly reduced and show a dose-dependent relation compared with hyperglycaemic (H group) mice. Similarly, the levels of total cholesterol, TAG and HDL-cholesterol in the serum of the Jerusalem artichoke inulin-treated group (LJ, MJ and HJ groups) were significantly lower than those in the hyperglycaemic group (group H) and show a dose-dependent relation. It indicated that Jerusalem artichoke inulin can reduce the FBG levels in hyperglycaemic mice and alleviate the abnormal blood lipid index caused by diabetes in hyperglycaemic mice (Table 4). Inulin decreased serum cholesterol and TAG concentrations, which is consistent with the previous findings^(44)^.

In total, forty-nine genes were up-regulated and thirty-five were down-regulated. Among the up-regulated and down-regulated ones, treatment differences were found in the expression of twenty-two genes including *LDLR*, *LDLRAP1*, *LRP6*, *STAB2* and *ANKRA2* as the LDL receptors^(45,46)^. *APOA4* is an LDL-related protein and is associated with cholesterol efflux, reversed cholesterol transport and cholesterol homoeostasis^(47,48)^. *CYP39A1* is related to cholesterol catabolism^(49)^. *TM7SF2*, *NSDHL and ACAA2* are related to cholesterol biosynthesis^(50,51)^ and *CYP7B1* to cholesterol metabolism^(52)^. *LCAT* is involved in HDL metabolism^(53)^.

The results of 16S rDNA gene sequencing indicated that the use of a HFD and STZ could change the composition of the intestinal microbiota of mice (online Supplementary Fig. S2). The mice intestinal microbiota improved (online Supplementary Figs. S3, S4 and S5) when treated with metformin HCl tablets, and a similar result was achieved with the Jerusalem artichoke inulin treatment. In particular, the inulin treatment increased intestinal abundance of Bacteroidetes and reduced intestinal abundance of Firmicutes (Fig. 3). These findings were consistent with the previous studies of inulin changing the abundance of intestinal bacteria. In the present study, the abundance of *Bacteroides* in the H treatment group was only 0·49 times that of the CK group. This is consistent with the published literature^(54,55)^. In contrast, the abundance of *Lactobacillus* in the H treatment group was 1·9 times that of the CK group. *Lactobacillus* is a type of ‘friendly’ bacteria that normally live in our digestive, urinary and genital systems, without causing disease. *Lactobacillus i*s used for treating and preventing diarrhoea and it is also used to treat high cholesterol, skin disorders, lactose intolerance, Lyme disease and hives and to boost the immune system^(56)^. *Bacteroides* is the predominant genus within the lower human intestinal tract, as evidenced by its prevalence in the product of this open-ended culture system, faeces. Within the intestinal tract, *Bacteroides* spp. host molecular interaction can influence host function, for example, in relation to immune system development^(57)^. Compared with the H group, the abundance of *Bacteroides* in the LJ and HJ inulin treatment groups increased significantly (2·4 and 9·1 times, respectively) (Fig. 4), which is consistent with the published results^(58)^.

### Conclusions

Jerusalem artichoke inulin reduced the levels of FBG and blood lipids in a dose-dependent manner and showed the antihyperglycaemic effects in mice fed the HFD and those treated with STZ. After the treatment with inulin, the expression of liver-related genes changed in the hyperglycaemic mice, and the proportion of *Bacteroides* in the intestinal tract of hyperglycaemic mice increased significantly. Jerusalem artichoke inulin may alleviate diabetes and increase the beneficial intestinal microbiota of HFD-fed hyperglycaemic mice and STZ-treated hyperglycaemic mice. Jerusalem artichoke inulin may be useful as a functional food ingredient in the prevention and/or treatment of hyperglycaemia.

## Supplementary material

For supplementary material accompanying this paper visit https://doi.org/10.1017/S0007114519002332. click here to view supplementary material
