# Supplementary material for: Inulin from Jerusalem artichoke tubers alleviates hyperglycaemia in high-fat-diet-induced diabetes mice through the intestinal microflora improvement
Source: Br J Nutr. 2020 Feb 14;123(3):308–18. doi: 10.1017/S0007114519002332 (PMC7015883; doi:10.1017/S0007114519002332)
Supplement: Supplementary file 1 [file S0007114519002332sup001.zip › S0007114519002332sup003.doc]

**Fig. S3.** Different structures of intestine microbiota in mice as influenced by the treatments CP and H (for the treatment notations see below). (A) Taxonomic representation of statistically and biologically consistent differences between the treatments. Differences are represented by the color of the most abundant class (red indicating CP, green indicating H and yellow indicating non-significance). The diameter of each circle is proportional to the abundance of a given taxonomic group. (B) The histograms of the LDA scores for differentially abundant taxonomic groups. The cladogram was calculated by LefSe and was displayed according to the effect size. CP, standard diet + 125 mg/kg metformin HCl tablets per day; H, standard diet + 5 g/kg physiological saline per day (experimental control group with induced diabetes).
