# Supplementary material for: Inulin from Jerusalem artichoke tubers alleviates hyperglycaemia in high-fat-diet-induced diabetes mice through the intestinal microflora improvement
Source: Br J Nutr. 2020 Feb 14;123(3):308–18. doi: 10.1017/S0007114519002332 (PMC7015883; doi:10.1017/S0007114519002332)
Supplement: Supplementary file 1 [file S0007114519002332sup001.zip › S0007114519002332sup006.doc]

**Table S1.** The compositions of the Standard (Control) Diet and High-fat Diet

| Standard Diet Indicator | Maintain feed (%) | High-fat Diet Indicator | MD12032  (45% fat kcal%)  (gm% / kcal%) |
| --- | --- | --- | --- |
| Moisture | ≤ 10.0 | Protein | 24 / 20 |
| Crude protein | ≥ 20.0 | Fat | 24 / 45 |
| Crude fat | ≥ 6.0 | Carbohydrates | 41 / 35 |
| Crude fiber | ≤ 5.0 | Trace element | 11 / 0 |
| Crude ash | ≤ 8.0 |  |  |
| Calcium | 1.0-1.8 |  |  |
| Phosphorus | 0.6-1.2 |  |  |
| Lysine | ≥ 1.32 |  |  |
| Methionine + cystine | ≥ 0.78 |  |  |
