# Supplementary material for: Inulin from Jerusalem artichoke tubers alleviates hyperglycaemia in high-fat-diet-induced diabetes mice through the intestinal microflora improvement
Source: Br J Nutr. 2020 Feb 14;123(3):308–18. doi: 10.1017/S0007114519002332 (PMC7015883; doi:10.1017/S0007114519002332)
Supplement: Supplementary file 1 [file S0007114519002332sup001.zip › S0007114519002332sup007.doc]

**Table S2.** Concentration of fasting blood glucose (FBG) in mice before and after the induction of type 2 diabetes by consuming high-fat diet feed (HFD-feed) and the streptozocin (STZ) treatment

(Mean values with their standard errors)

| Concentration (mmol/L) | FBG | |
| --- | --- | --- |
| Designated treatment groups | Before the induction | After the induction |
| CK^⸹^ | 8.05±0.30^a^ | 6.40±0.18^b^ |
| H^⸸^ | 8.04±0.36^a^ | 14.98±1.00^a^ |
| CP^⸷^ | 8.48±0.26^a^ | 14.30±0.33^a^ |
| LJ^*^ | 8.56±0.27^a^ | 13.70±0.71^a^ |
| MJ^§^ | 8.79±0.32^a^ | 14.85±0.88^a^ |
| HJ^‡^ | 7.93±0.32^a^ | 14.83±0.68^a^ |

^a,b,c^ Values in the same column not sharing a common superscript are significantly different (p≤0.05).

^⸹^CK, standard diet + 5 g/kg physiological saline per day (blank control).

^⸸^H, standard diet + 5 g/kg physiological saline per day (experimental control group with induced diabetes).

^⸷^CP, standard diet + 125 mg/kg metformin HCl tablets per day.

^*^LJ, standard diet + 2.5 g/kg inulin per day.

^§^MJ, standard diet + 5 g/kg inulin per day.

^‡^HJ, standard diet + 10 g/kg inulin per day.

The CK group of mice was fed standard diet and given citrate buffer vehicle; the other five groups of mice were fed HFD and injected intraperitoneally with low dose of STZ to induce type 2 diabetes before the start of inulin treatments.

All mice had unlimited access to drinking water.
